# Urban heat island conditions experienced by the Western black widow spider (*Latrodectus hesperus*): Extreme heat slows development but results in behavioral accommodations

**DOI:** 10.1371/journal.pone.0220153

**Published:** 2019-09-06

**Authors:** J. Chadwick Johnson, Javier Urcuyo, Claire Moen, Dale R. Stevens

**Affiliations:** 1 School of Mathematics and Natural Sciences, Arizona State University at the West Campus, Glendale, AZ, United States of America; 2 Department of Biology, Lasry Center for Bioscience, Worcester, MA, United States of America; National Institute of Biology, SLOVENIA

## Abstract

While shifts in organismal biology stemming from climate change are receiving increased attention, we know relatively little about how organisms respond to other forms of anthropogenic disturbance. The urban heat island (UHI) effect describes the capture of heat by built structures (e.g. asphalt), resulting in elevated urban temperatures. The UHI is a well-studied phenomenon, but only a handful of studies have investigated trait-based shifts resulting from the UHI, and even fewer have attempted to quantify the magnitude of the UHI experienced at the microclimate scale. Here, using a common urban exploiter, the Western black widow spider (*Latrodectus hesperus*), we show that the UHI experienced by spiders in July in their urban Phoenix, AZ refuges is 6°C hotter (33°C) than conditions in the refuges of spiders from Sonoran Desert habitat outside of Phoenix’s development (27°C). We then use this field microclimate UHI estimate to compare the development speed, mass gain and mortality of replicate siblings from 36 urban lineages reared at ‘urban’ and ‘desert’ temperatures. We show that extreme heat is slowing the growth of spiderlings and increasing mortality. In contrast, we show that development of male spiders to their penultimate moult is accelerated by 2 weeks. Lastly, in terms of behavioral shifts, UHI temperatures caused late-stage juvenile male spiders to heighten their foraging voracity and late-stage juvenile female spiders to curtail their web-building behavior. Trait-based approaches like the one presented herein help us better understand the mechanisms that lead to the explosive population growth of urban (sometimes invasive) species, possibly at the expense of urban biodiversity. Studies of organismal responses to the present day UHI can be used as informative surrogates that help us grasp the impact that projected climate change will have on biodiversity.

## Introduction

The impact of human activity on native biodiversity is a critical question that needs to be quantified to minimize the damage we do to ecosystems [1,2]. The impact that ‘Human Induced Rapid Environmental Change’ (HIREC) has on ecosystems has unknown consequences for the long-term sustainability of the ecosystems that humans inhabit and rely upon [3,4]. Urbanization is a particularly important example of HIREC, as 70% of all humans are expected to live in rapidly expanding urban centers by 2050 (United Nations, 1996). While studies have shown that urbanization can often benefit certain species at the expense of others [5], causing an overall decrease in biodiversity [6], more studies need to identify the organismal drivers (e.g. physiological mechanisms and/or behavioral traits) that allow some species to proliferate in the face of anthropogenic change [7,8], but see [9]. Furthermore, the field of urban organismal ecology has been dominated in the past by studies of vertebrates (in particular, urban birds, e.g. [5,6]) and has been slower to address the importance of urban arthropods (including the loss of species diversity) in the urban ecosystem [10]. For example, Hemipteran pests seem to thrive as urban environments heat up, developing significantly faster than they do in cooler, less disturbed environments. In contrast, the parasitoids that help limit Hemipteran populations do not experience a similar shifted phenology, thus further promoting Hemipteran population growth in disturbed habitats [11]. Despite the growing number of studies showing the profound impacts of human disturbance on arthropod populations, much work is still needed to address the fundamental question of why some arthropods thrive in urban ecosystems, and how that success may compromise urban biodiversity.

One research focus that has emerged examines the ways in which urban temperature increases influence organisms [12]. While studies of global climate change typically must rely on simulations of future climate scenarios, and their projected temperature increases, work on urbanization can take advantage of the fact that urban areas allow us to study now the temperature increases that global climate change is predicted to bring in the future [12]. Many studies address the dynamics of elevated temperatures in cities, due in large part to the urban heat island (UHI) phenomenon. Urbanization drives the UHI through the elimination of vegetative cover and the addition of anthropogenic heat sources (e.g. automobiles and impervious surfaces) [13]. These impervious surfaces capture heat during the day and can cause urban habitats to be up to 10°C hotter than surrounding areas [14]. The UHI effect may be especially important to understand in extreme ecosystems such as deserts, where abiotic factors (e.g. temperature, water limitation) and biotic resources (e.g. prey limitation) present particularly strong selective forces.

While UHI temperature increases are likely to impact all organisms, urban arthropods are a surprisingly understudied group that would seem to be particularly influenced by the elevated temperatures of the UHI. Arthropods are critical players in the ecosystem—providing vital ecosystem services (e.g. seed dispersal, pollination, decomposition, predation) making them crucial to the functioning of urban ecosystems [15]. Arthropods are likely influenced by the UHI because of both their ectothermic reliance on external temperature cues for their life history and the physiological effect increased temperature has on their metabolic chemical reactions. Specifically, increased urban heat could benefit urban arthropod populations to the extent that it speeds metabolism and shifts phenology (e.g. [16]). Alternatively, if UHI conditions exceed critical, species-specific thresholds, then urban arthropods will be poorly equipped to tolerate the disturbed microclimate and unlikely to persist. For example, variation in urban landscaping can severely limit urban lizard activity, and presumably fecundity and population growth [17]. As for arthropods, in a forest warming experiment, ant species with higher thermal tolerances showed increased nest occupancy relative to species less capable of tolerating extreme heat [18]. Also, most arthropod families in higher latitude cities benefit from UHI temperatures, though this expected response was present in some cities, but not in others—suggesting that urbanization involves much more than just the UHI [19,20].

Human impacts on the environment, such as the UHI, can have dramatic impacts on ecology at the species level, and such community dynamics are presumably shaped by the individual organism’s ability to behaviorally and physiologically tolerate HIREC (e.g. see [7,9,16]). For example, high levels of urbanization in Belgium favor Carabid beetle species that can i) disperse easily, and ii) tolerate high temperatures [21]. More generally, urbanization affects a wide-ranging set of behaviors (e.g. activity, escape response, aggression) from a variety of taxa (e.g. mammals, birds, arthropods [9,22]).

Here we address the effects of UHI temperatures on the development and behavior of Western Black Widow spiders, *Latrodectus hesperus*, a superabundant urban exploiter found across western North America. *L*. *hesperus* thrive in urban habitats, often infesting urban parks, schools, industrial settings, and residences at population densities that dwarf their counterparts in Sonoran desert habitat [23]. We focus our work on black widow populations across the urbanized Sonoran Desert, specifically the metropolitan area of Phoenix, AZ. Phoenix is routinely one of the fastest growing cities in the U.S. with a greater metropolitan population of 1 million in 1970, now exceeding 4.5 million [13].

The UHI has been relatively well studied in Phoenix [13,24], including some treatment of the impact of the UHI on vertebrate biology [17]. Generally, Phoenix experiences a 3°C UHI relative to the surrounding Sonoran Desert, though much variability exists across different types of urban land use [13,25] and studies rarely quantify the UHI experienced at the microclimate scale experienced by different urban organisms (but see [26]). A recent review suggests that any understanding of organismal responses to global change (especially ectotherms inhabiting urban habitats) will need to address habitat variables from a local (microclimate) level [27]. Specifically, recent work on the thermal biology of lizards suggests that much complexity found in the urban ecosystem (e.g. socioeconomics, patch size, UHI, water use, land cover …) determines the persistence of urban lizards [17,26].

Here, we test the hypothesis that UHI conditions should have profound influences on the life history of an ectothermic urban arthropod. First, isolating the known urban and desert microclimates inhabited by black widows, we predict urban microclimates will be significantly hotter. Second, assuming these temperature increases favor an urban ectotherm, we predict increased development speed. Alternatively, if the temperature differences we find have surpassed a critical threshold, UHI conditions could lead to slowed development, reduced mass and increased mortality. Finally, we predict UHI conditions will force spiders to heighten web building and voracity towards prey in an attempt to compensate for the heightened metabolism that comes with increased temperatures.

## Materials and methods

### Establishment of Phoenix’s urban heat island experienced by black widows

Field studies did not involve work on endangered or protected species. Across the month of July 2016, iButtons recorded temperature every 5 minutes from the refuge of a single adult female black widow from each of four urban collecting locales (lat.-long.: 33.597256–112.180383, 33.561977–112.180830, 33.567907–112.124740, 33.627612–112.151331) and four desert collecting locales (lat.–long.: 34.130961–112.088018, 34.113567–112.355665, 33.402360–111.350040, 33.500475–111.458843). These sites were always separated by a minimum of 10 km. Urban measures/spider refuges came from residential habitat (e.g. park benches, hedgerows lining schoolyards, cracks in concrete block walls), whereas desert measures/spider refuges came from undisturbed Sonoran Desert habitat a minimum of 15 km outside of Phoenix’s urban growth. Collection sites were found on either unprotected public lands, or private lands where the land owner had given us permission to work.

### Egg/spiderling development

Adult female *L*. *hesperus* were collected from 10 urban Phoenix sites similar to those described above. Spiders were housed individually in boxes measuring 10×10×12 cm. On the day of its deposition (day 0), egg sacs were collected from 36 females representing these 10 urban collection sites. No later than day 3, 50 eggs from each family were weighed (μg, Cahn Microbalance) and egg area was digitally imaged (mm^2^). Eggs (n = 1800) were then housed individually in boxes measuring 4×4×6 cm with a piece of cotton lining the bottom and two toothpicks crossing diagonally to provide a structure for eventual web building. These boxes were initially housed at 24°C on a 12:12 light-dark cycle. *L*. *hesperus* is native to desert habitats and as such can be reared in the laboratory with no augmentation to humidity. Beginning day 30 of development, each spiderling was fed two *Drosophila melanogaster* twice a week. While *L*. *hesperus* spiderlings are highly cannibalistic [28], we have never seen cannibalism happen prior to day 30 (Johnson, unpublished data). Each box was checked daily for molts and deaths. On day 44 of development, each family was divided evenly into incubators simulating urban (33°C) and desert (27°C) temperature conditions (see Results below). We waited until day 44 to begin the UHI treatment so as to allow spiderlings a chance to build web and feed before stressing them with extreme heat. Incubators were held on a 12:12 light-dark cycle. At day 105, all surviving spiderlings were weighed (mg).

### Male development and behavior

Given that males have a much shorter lifespan than females, we continued to follow males through their final juvenile molt (‘penultimate’ molt). Male food regimes were doubled for their penultimate molt (i.e. 4 flies twice weekly), and male feeding voracity (latency to kill prey in seconds) was scored during these feedings. Specifically, a spider’s voracity was scored as the latency between the release of prey in the middle of the web and the time a spider initiated silk wrapping. The development study ended when we recorded each male’s adult molt date, as very few males from the urban heat treatment survived this final molt (see Results).

### Female web building

In a separate experiment we examined the effect of urban heat conditions (27 vs. 33°C) on the web building behavior of 36 female black widows across their final two juvenile molts and into adulthood. To clarify, we examined these effects using lineages separate to those described above. For these new lineages, females hatched in the laboratory from urban parents were raised at 24°C until the antepenultimate molt, at which point they were randomly assigned to one of the two temperature treatments, urban or desert, as defined above. Spiders were fed a standard-sized House cricket (*Acheta domesticus*) 7 days before each web-building trial. Females were housed in a walk-in chamber for 7 days at their prescribed temperature treatment in individual boxes measuring 10×10×12 cm. At the end of those 7 days, spiders were weighed (mg) and returned to their respective temperature treatment in a large, clean web-building tub (57×38×33 cm.). These tubs were equipped with sandy substrate and a crisscrossed pair of wooden dowels that led from the floor up into a refuge made out of a crevice inside of a Styrofoam wall (see Fig 1 of [29]). After a 10-minute acclimation period, we scored spiders as web building or inactive every 10 minutes for the first 3 hours of their dark cycle. Following a trial, spiders were returned to their smaller boxes, fed a cricket, and then reassigned to the temperature treatment (chamber) they did not experience previously. Web-building tubs were wiped with alcohol before each new trial and sand, dowels and Styrofoam were replaced for each new trial. This temperature pairing was repeated 7 days following the ante-penultimate, penultimate and adult molts.

### Statistical analysis

Because our work has previously shown that many parts of the black widow’s life history (e.g. egg size, development speed, cannibalism, dispersal) are characterized by a significant family effect [28,30,31]), we included family (n = 34) as a random factor in our ANOVAs designed to test for an effect of temperature on spider development and behavior. For female behavior, we exposed each female to both temperature treatments (order randomized) and used paired t-tests to look for an effect of temperature within developmental stages, and a repeated measures ANOVA to look for effects across developmental replicated assays.

## Results

**Fig 1:**
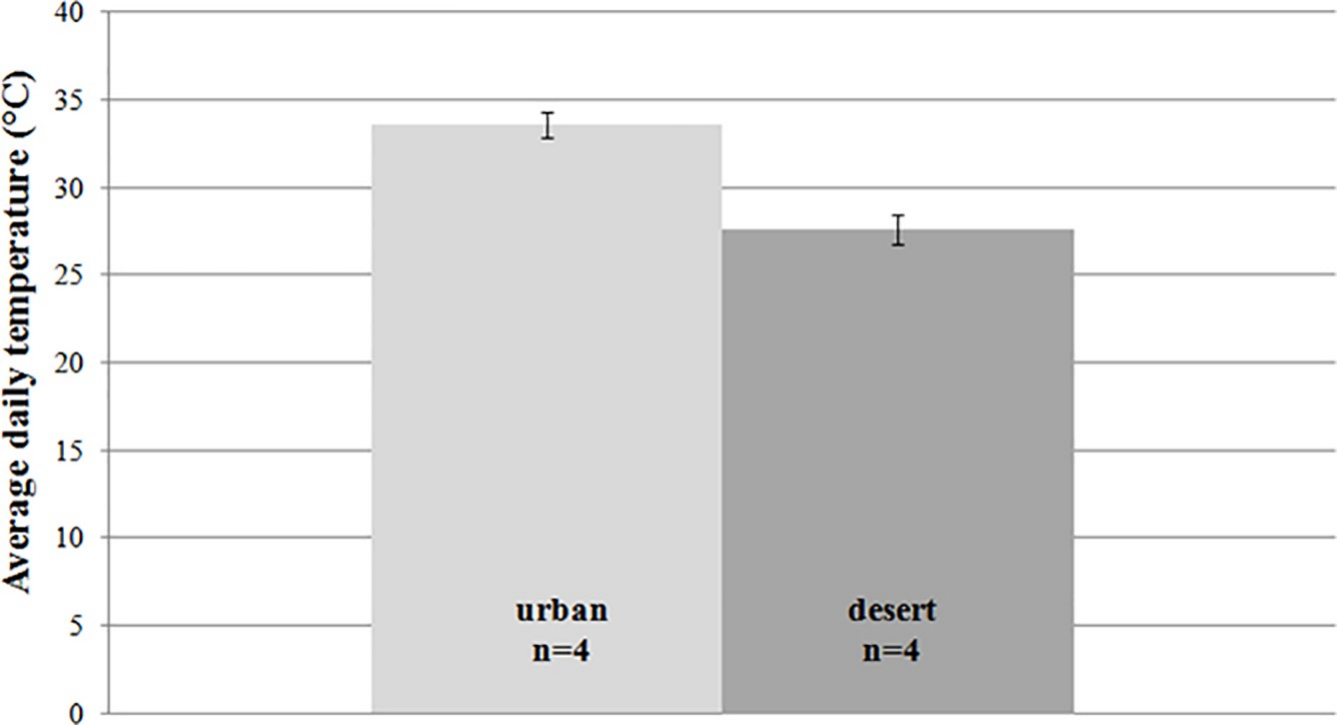
The UHI experienced in black widow refuges results in a 6°C temperature elevation.

**Fig 2:**
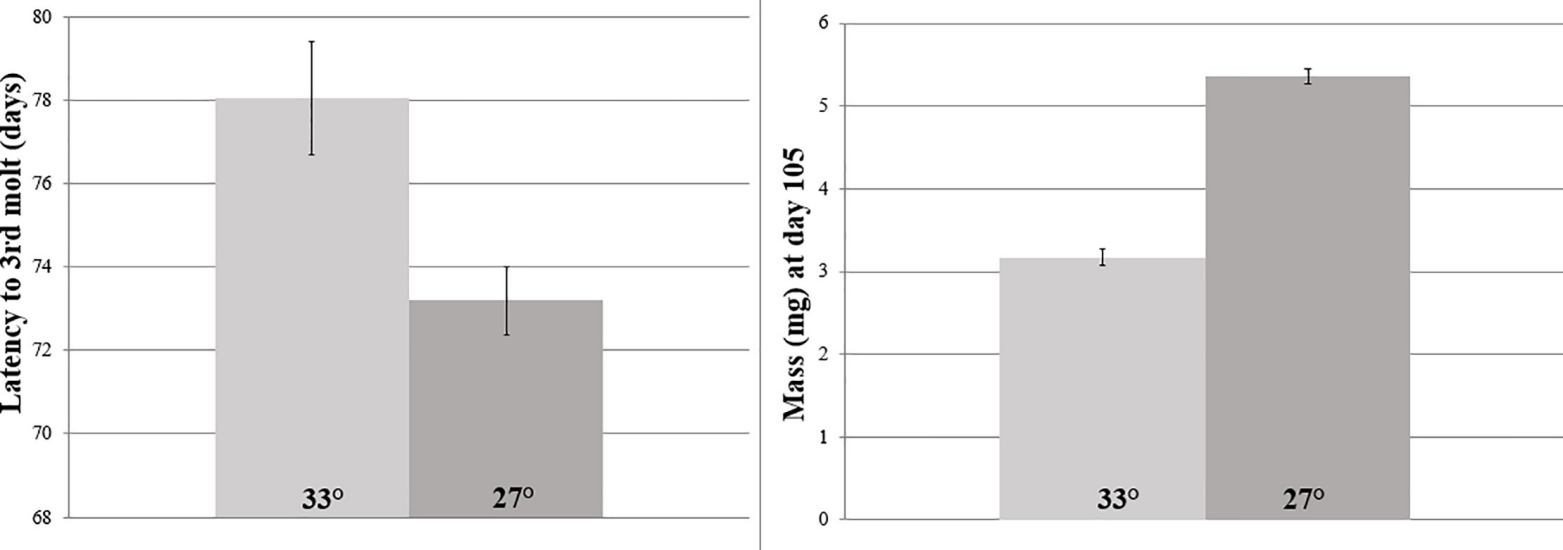
UHI temperatures significantly slow development and reduce body mass of black widow spiderlings as they approach the 3^rd^ molt.

From iButtons, we took the daily average temperature across July 2016 for four desert and urban black widow microclimates and found that the UHI yields a 6°C temperature elevation (desert = 27 vs urban = 33°C; F_1,6_ = 43.2, p = 0.001; see Fig 1). Looking at the effect of this temperature difference on early spiderling development to the 3^rd^ molt (see Fig 2), we found that family had a highly significant effect (F_33,1010_ = 254, p<0.0001), UHI temperatures slowed development by 5 days (approximately 25% of the interval between molts 2 and 3; F_1,1010_ = 40.4, p<0.0001), and there was no significant interaction between family and temperature (F_33,1010_ = 1.15, p = 0.26). Similarly, spiderling mass at day 105 was affected by family (F_33,755_ = 3.28, p<0.0001), reduced by UHI temperatures by more than 2 mg (almost a 50% difference; F_1,755_ = 531, p<0.0001) and there was a significant interaction between family and temperature (F_33,755_ = 2.90, p<0.0001; see Fig 2). As for the longevity of spiderlings that survived at least to molt 3, family showed a non-significant effect (F_33,1010_ = 3.64, p = 0.091), UHI temperatures cut spiderling longevity by more than 50% (F_1,1010_ = 126.9, p<0.0001), and there was no significant family × temperature interaction (F_33,1010_ = 1.50, p = 0.191).

**Fig 3:**
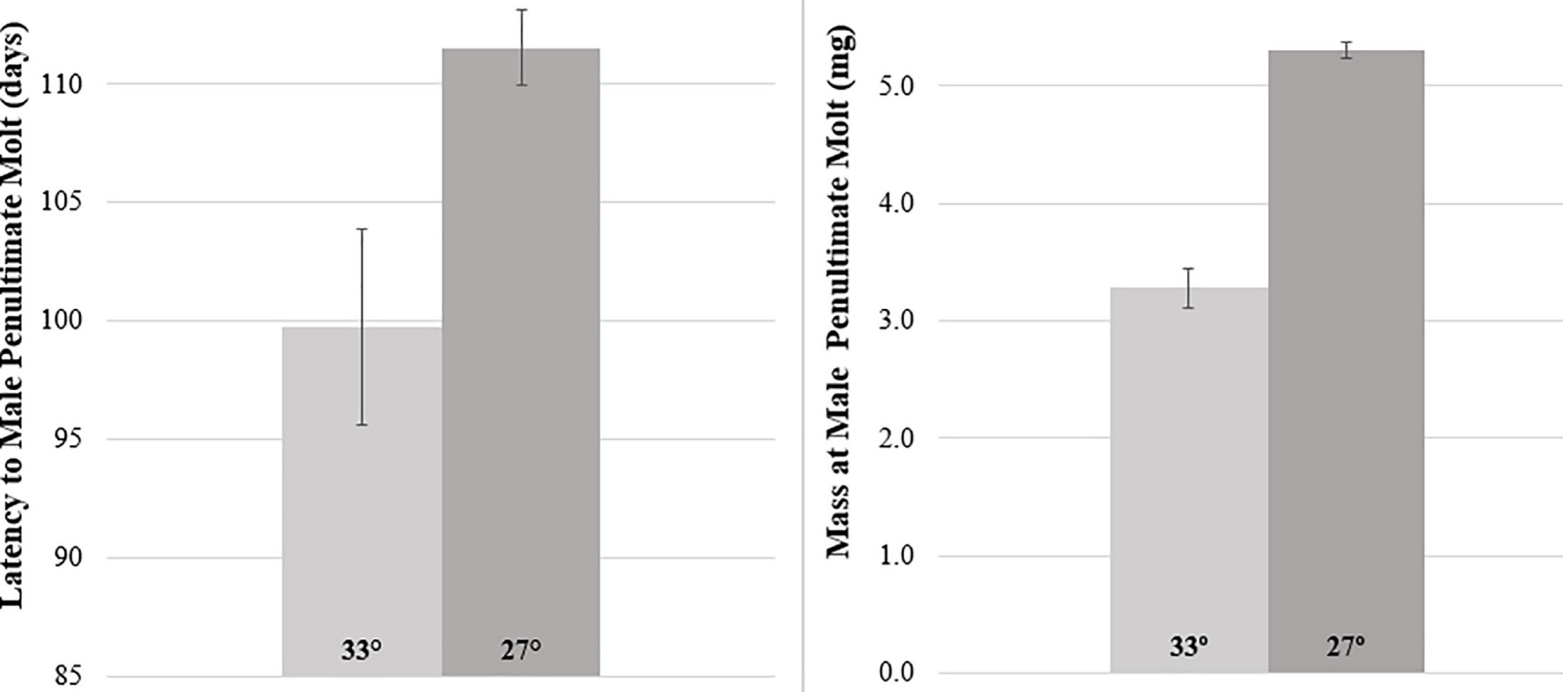
UHI temperatures significantly speed male development to the penultimate molt while still significantly reducing body mass.

In contrast to the slowed development observed in early juveniles in the urban heat group described above, the urban heat treatment accelerated male development to the penultimate molt by almost 2 weeks, a result that was not statistically significant (F_1,308_ = 1.61, p = 0.222; Family:

F_33,308_ = 4.49, p = 0.043; Family × Temperature: F_12,308_ = 1.61, p = 0.222; see Fig 3). Male development speed to the adult molt was not analyzed as only a single male experiencing urban heat conditions completed this molt, and he died the following day. UHI temperatures reduced male mass at the penultimate molt by 40% (F_1,262_ = 61.1, p<0.0001; Family: F_33,262_ = 1.21, p = 0.41; Family × Temperature: F_12,262_ = 1.59, P = 0.094; see Fig 3). Accordingly, male longevity was significantly shortened by the UHI treatment (F_1,262_ = 59.3, p<0.0001; Family: F_33,262_ = 1.29, p = 0.40; Family × Temperature: F_12,262_ = 1.29, P = 0.110).

**Fig 4:**
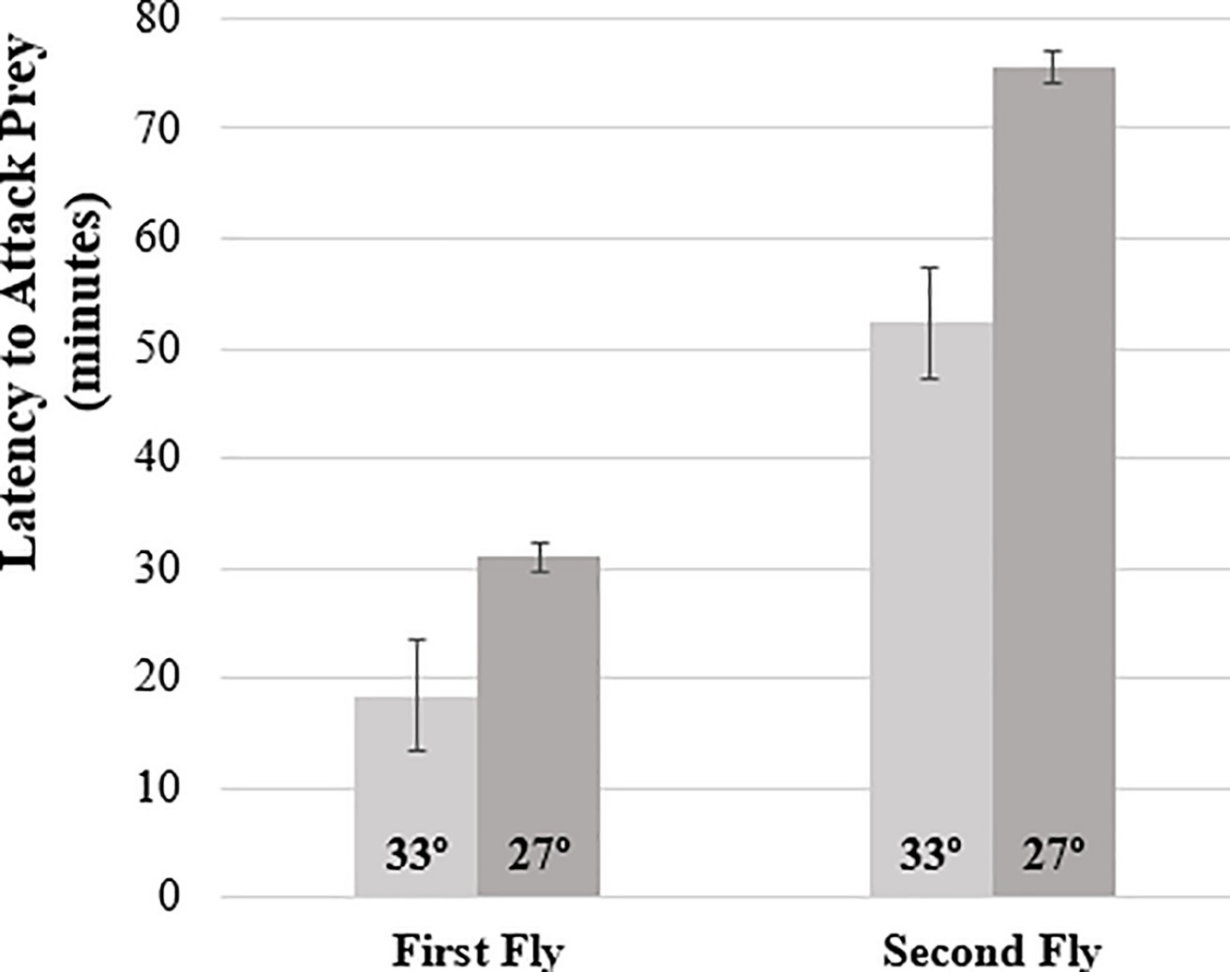
Penultimate molt males reared at UHI temperatures compensate for reduced mass by significantly increasing their aggression towards prey items.

Perhaps to counter the metabolic demands and reduced mass seen for urban heat conditions, penultimate molt males from the 33°C treatment attacked both their first fly (F_1,294_ = 9.65, p = 0.006; Family: F_33,294_ = 7.37, p = 0.110; Family × Temperature: F_12,294_ = 0.49, p = 0.917) and second fly (F_1,295_ = 17.6, p = 0.001; Family: F_33,294_ = 3.12, p = 0.069; Family × Temperature: F_12,295_ = 1.06, p = 0.393; see Fig 3) significantly faster than 27°C siblings (Fig 4).

**Fig 5:**
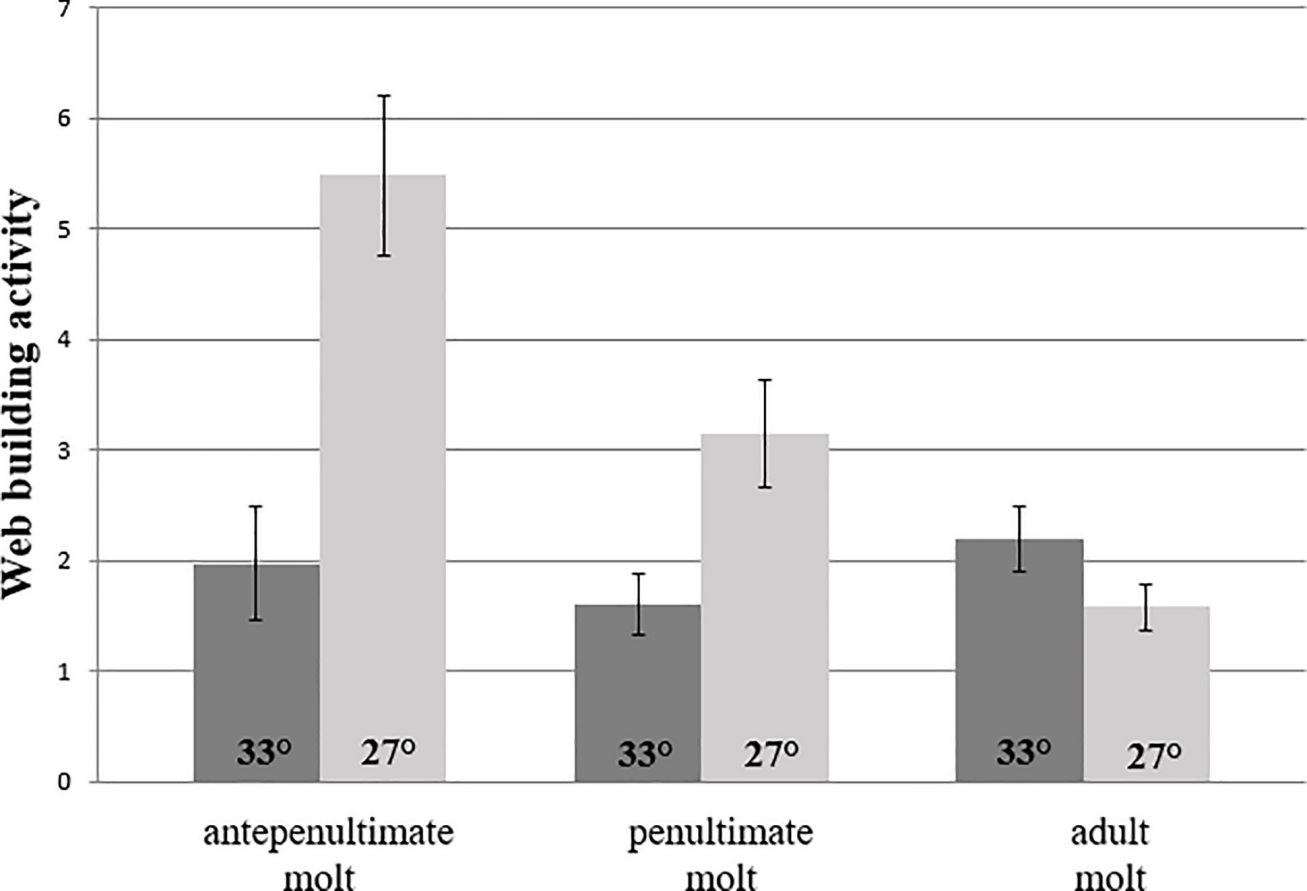
UHI temperatures dramatically reduce web building behavior by females in their final two juvenile molts, an effect that is lost at adulthood.

Lastly, the time spent building web was significantly reduced in urban heat conditions for females in the two molts preceding adulthood (ante-penultimate molt: t = 5.2, d.f. = 32, p<0.0001; penultimate molt: t = 3.0, d.f. = 32, p = 0.005, see Fig 5). This difference was not present for adult female web-building behavior (t = 1.89, d.f. = 30, p = 0.07). Repeated measures ANOVA showed that when females experienced UHI temperatures, their low level of web building behavior did not change across development (F_2,60_ = 0.37, p = 0.69), but when they experienced the 27°C treatment, high levels of juvenile web building were significantly reduced at adulthood (F_2,60_ = 13.06, p<0.0001, see Fig 5).

## Discussion

The black widow is experiencing a dramatic 6°C UHI effect in the disturbed Sonoran desert habitat of Phoenix, AZ—an effect that is double the general estimates for Phoenix that do not take into account the microhabitat that urban organisms experience [13]. We find that such a temperature elevation likely surpasses a critical threshold for this species (somewhere between 27 and 33°C) that has yet to be isolated. UHI temperatures significantly slowed development, reduced body mass and increased mortality of early instars. In contrast, UHI temperatures sped up development to the male’s penultimate molt (but still at reduced body mass) resulting in males approaching maturity at a relatively small body size. Subsequently, males reared at UHI temperatures were more aggressive towards prey during their penultimate molt, but at the prey levels we offered in this experiment were unable to sufficiently compensate and experienced almost complete mortality at the adult molt. Lastly, ante-penultimate and penultimate molt females were significantly less active building web at 33°C than they were at 27°C. This effect was lost at adulthood when web building activity was drastically reduced for females in the 27°C treatment.

### Phoenix’s urban heat island experienced by black widows

Any attempt to understand UHI impacts on organismal biology and biodiversity will require fine scale assessments of the thermal environments being experienced by the biota of interest [27,32]. As noted above, the 6°C UHI shown here for black widow microclimates is double the general UHI estimate for urban Phoenix daily averages taken from weather station measures of air temperature [13]. Organisms that rely on heat-retaining urban infrastructure for their livelihoods (e.g. cracks in concrete block walls serving as ideal spider refuges) may be experiencing a much more drastic UHI than we have previously assumed.

While such microclimate UHI data are a necessary starting point for understanding the implications of the UHI for organisms, we note several instructive caveats to our estimates. First, we purposely sampled an extreme summer month (July) and presented experimental spiders with the average temperature from urban and desert microhabitats. As such, the present experiment fails to capture 1) the variation in UHI that is certainly present across the breeding season (e.g. early and late urban breeders may experience a milder UHI than the mid-summer UHI modelled here), and 2) the variation that is certainly present within a 24-hour period. This latter circadian variation can only be experimentally imposed when researchers have access to temperature chambers that allow for hourly temperature fluctuations, but this would seem critical moving forward as using daily averages means presenting animals with daytime temperatures that are lower than field measurements, and nighttime temperatures that are higher than field measurements [20]. An organism’s performance may be more dramatically impacted by variation in temperature than it is by mean temperature [33], and evidence exists to suggest that the mean and variance in temperature interact to impact fitness [34].

Another issue that complicates any estimate of UHI effects on an organism’s performance is the degree to which microclimate either buffers or amplifies environmental conditions [32]. Given that our data suggest this species would struggle to survive in the city at these UHI temperatures, and yet black widows are, in fact, thriving in Phoenix, it seems likely that spiders are finding some buffer from these extreme conditions. First, black widows employ a nocturnal foraging strategy that is common for desert organisms faced with extreme daytime conditions. As such, spiders emerge from refuge in the evening when temperatures drop and remain in refuge during the light phase. However, the UHI is best known for its capture of daytime heat and resultant increase of nighttime temperatures [24,35]. Interestingly, urban black widows appear to emerge near dusk when nighttime temperatures are their highest and return to refuge several hours later, well before nighttime temperatures have dropped to their absolute lows. We might conclude from this that spider activity is more influenced by prey activity than by nocturnal temperature variation. Regardless, while the nocturnal foraging behavior of urbanblack widows buffers them from extreme daytime temperatures, there is no evidence that urban black widow activity is driven by buffering UHI nighttime temperature extremes. The black widow’s use of early evening hours for foraging exposes them to the most extreme nighttime temperatures.

Additionally, we have a poor understanding of the dynamics of refuge use in these (and most) spiders. For example, our iButtons were placed inside of the spider’s refuge, but spiders may choose highly complex refuges that provide a temperature gradient. Females actively guard their egg sacs both out in the web and inside of the refuge (Johnson, pers. obs.). If females are moving deeper inside a complex refuge with their egg sacs during extreme heat, they could successfully buffer eggs from extreme heat and optimize egg/larval development speed and survival. While such a prediction would be challenging to test, it would be useful to investigate a potential relationship between evening temperatures and the location of egg sacs relative to refuges in the web.

Lastly, it is likely that spiderlings emerging from the egg sac are capable of behaviors that buffer them from extreme UHI conditions. While it is typical of emergent spiderling clusters to inhabit the maternal web for several weeks, opportunistically feeding (e.g. cannibalizing each other or taking other small prey) urban spiderlings may instead be driven to remain in the refuge and/or disperse prematurely to optimally thermoregulate. Such dramatic shifts to the urban life history would seem likely to carry fitness costs as emergent spiderlings cannot, at first, build webs of their own that are large enough to capture large prey. Future studies will examine any tendency for urban black widows to express a philopatric failure to disperse from the maternal habitat, potentially resulting in decreased gene flow.

### Development

A full understanding of urban success requires a focus on the organismal drivers (mechanisms) that facilitate growth, survival and reproductive success in highly disturbed environments [7,8]. Here we have shown that our estimates of the black widow’s UHI have only negative impacts on these fitness surrogates. Theory suggests that species already living close to their thermal maximums will experience the greatest fitness declines from elevated temperatures [36]. Indeed, the developmental and survival costs of 33°C seen here would likely doom an urban population of black widows in the absence of some sort of buffer or refuge from the UHI conditions suggested above. Understanding the complexity of urban disturbances (e.g. how the UHI varies at different spatial scales) and how different species respond differently to such disturbances is a major challenge for researchers [37].

Perhaps most simply, it may be that the supplemental water and abundant urban prey seen in Phoenix, AZ, [15] but not accounted for in our experiments, allows urban spiders to endure metabolic costs imposed by UHI conditions. For example, during seasonal drought periods, water-supplemented Gila monsters (*Heloderma suspectum*) stayed better hydrated and were more active in searching for prey than lizards receiving no added water [38]. It remains possible that a treatment that imposed UHI temperatures, while at the same time providing prey abundance typical of urban habitats, may have been beneficial—resulting in faster developing spiders that feed sufficiently to maintain the mass required to survive to adulthood.

### Behavior

Our temperature manipulations had sex-specific effects on spider behavior. Lifelong UHI rearing conditions stimulated penultimate molt males to increase their voracity towards prey. In contrast, 7 days of urban heat caused late-stage juvenile females to curtail their web building behavior. This result is consistent with the anecdotal observation that urban black widow webs are often dwarfed in size compared to the webs made by spiders inhabiting Sonoran Desert habitats (Johnson, pers. obs.). This suggests that black widows are capable of the plasticity required to survive in rapidly changing urban environments (see also [29]). These contrasting effects for males and females make sense if we consider the short life history trajectory of males compared to the longer trajectory of females. It seems reasonable that urban heat could increase male activity and voracity to ensure they survive their brief juvenile development to have a chance at mating, whereas females experiencing urban heat (especially under high levels of urban prey abundance) can afford to reduce activity and web building behavior. Such a rationale is reinforced by the general lack of urban enemies (e.g. wasp parasites of egg sacs, Johnson, unpublished data), which releases urban widows from the pressure to build large webs for protection.

## Conclusions

We offer a relatively rare data set, which directly quantifies and then manipulates a known variable stemming from urbanization to ask whether that variable impacts development and behavior in urban organisms. Thus, we are moving past the ‘low-hanging fruit’ that has dominated urban evolutionary ecology in the past [7]. The documentation of a microclimate UHI effect that is double that suggested for urban Phoenix at a large scale [13], and triple the estimates of recent climate change predictions in the absence of the UHI (IPCC, 2014) suggests that urban organisms are likely experiencing dramatic temperature elevations, and more work needs to be done to understand the implications of such shifts. In particular, such studies will help us better understand the mechanisms that lead to the explosive population growth of urban (sometimes invasive) species, possibly at the expense of urban biodiversity. More generally, studies of organismal responses to the present day UHI can be used as informative surrogates that help us grasp the impact that projected climate change will have on biodiversity.
